# The relationship between tolerance for uncertainty and academic adjustment: the mediating role of students’ psychological flexibility during COVID-19

**DOI:** 10.3389/fpsyg.2023.1272205

**Published:** 2023-11-17

**Authors:** Esma Daşcı, Kübra Salihoğlu, Esra Daşcı

**Affiliations:** ^1^Independent Researcher, Ankara, Türkiye; ^2^Psychological Counselling and Guidance, Department of Education, Istanbul Üniversitesi-Cerrahpaşa, Istanbul, Türkiye; ^3^Educational Psychology, Department of Education, Kastamonu University, Kastamonu, Türkiye

**Keywords:** COVID-19, intolerance for uncertainty, psychological flexibility, online academic adjustment, wellbeing

## Abstract

**Introduction:**

University students are among the groups most adversely affected by COVID-19 in terms of their psychological and academic wellbeing, particularly given the pandemic’s uncertainty. However, little is known about their psychological flexibility to deal with this uncertain nature of pandemic. This study aimed to examine the mediating role of psychological flexibility (PF) in the relationship between university students’ intolerance of uncertainty (IU) and their academic adjustment to the online learning process during COVID-19.

**Methods:**

Data was collected from 388 university students from Türkiye (18–34 (*M*_*age*_ = 21.43, *SD* = 2.04) who completed five questionnaires – Intolerance of Uncertainty Scale, Acceptance and Action Questionnaire II, Academic Self Efficacy Scale, Educational Stress Scale, and Online Self-Regulatory Learning Scale. Additionally, as indicators of students’ academic adjustment, perceived academic performance – ranging 1 to 10, and their last academic grade point average before and during the pandemic were also collected.

**Results:**

The results indicated that PF and IU had a significant effect on the academic adjustment of students during the pandemic. PF had a complete mediating role between the IU and academic adjustment. Results also revealed that online self-regulation, a sub-dimension of academic adjustment, did not show a significant relationship with any other variable in the model.

**Discussion:**

Research findings showed that psychological flexibility is a very important strength for university students to maintain their academic adjustment in stressful times. The results were discussed in light of the relevant research, and recommendations for further research and implications were provided.

## 1. Introduction

Everyone’s daily habits had to abruptly shift due to the coronavirus pandemic (COVID-19), which shocked and alarmed everyone. Social isolation, public mask use, and lockdowns were only a few of the significant measures governments took to mitigate the infection. The lockdowns, in particular, were one of the most significantly altering mitigation measures for the global populace since they made everyone drastically shift their everyday schedules and behaviors. According to studies, the lockdown was one of the most challenging aspects of the pandemic because it immediately affected people’s physical and, by extension, psychological wellbeing (Thakur et al., 2020; Lim and Pranata, 2021). Rapid and unanticipated restrictions on people’s activities during the epidemic created both psychological and economic uncertainty as people were unsure about the immediate future (Baker et al., 2020). The pandemic caused a lot of people to worry about losing their jobs and income, which added to the feeling of uncertainty (Zavras, 2021). Along with the rapid change in routines and economic status, people’s psychological wellbeing was impacted by elevated death rates and existential threat (Satici et al., 2020b). To completely comprehend what happened, all of this novel and unexpected information needed to be unpacked and empirically investigated. Therefore, the current study’s goal is to investigate coping strategies and academic adjustment during COVID-19 by putting university students as its center of attention.

Numerous studies on the psychological symptoms people experience during the COVID-19 process have shown that people suffer from a variety of psychological issues, including anxiety, depression, stress, and sleep issues, and that their level of life satisfaction has significantly decreased as a result (Deng et al., 2020; Hyland et al., 2020; Özdin and Bayrak Özdin, 2020; Salari et al., 2020). Throughout this process, people struggle to find a way to fit into their new daily routines and deal with the psychological issues they encounter. Although everyone has dealt with everyday worry and anxiety because of the pandemic, university students’ burden was particularly complex. As a significant portion of the population, university students undoubtedly represent one of the demographics that are impacted by this future insecurity. During the outbreak, university students—who make up around 10% (8 million) of Türkiye’s population—continued their education online. While going through this novel mode of education, they had to deal with the epidemic’s fear and worry as well as the uncertainty around the direction their future professional life might take. Indeed research has indicated that the pandemic has a detrimental effect on university students’ mental health (Wang et al., 2020; Copeland et al., 2021; Gadosey et al., 2022; Birmingham et al., 2023). For instance, a study conducted with Turkish university students indicated that the degree of intolerance for uncertainty was linked to various detrimental effects on mental health, such as depression, anxiety, and stress (Şentürk and Bakir, 2021). Additionally, s tudents at universities who are attempting to maintain their academic commitments as well as the anxiety and despair they suffer throughout the epidemic are experiencing a variety of anxiety and stress issues as a result of the pandemic. In their study, Aristovnik et al. (2020) found that students’ perceptions of their own success in the online learning environment are negative due to their perceptions of inadequate computer skills and an abundance of homework; they are concerned about what they need to do for their future professional careers. Additionally, they stated that they experienced intense anxiety, boredom and disappointment. This data shows that most students seem to have a poor experience with online education. Therefore, it is crucial to investigate how students’ psychological flexibility to uncertainty during online learning influences their academic adjustment.

### 1.1. Academic adjustment

Given the strain, anxiety, and sense of helplessness felt by university students during the epidemic, it is quite possible that the uncertainty brought on by the epidemic has an effect on their ability to function in their daily lives, particularly in terms of their academic adjustment. The accomplishment of the obligations and responsibilities of the students related to the educational level they are in is known as *academic adjustment*. While academic achievement is crucial for the growth of abilities and the acquisition of new skills, a student’s ability to adjust to university life also has a bearing on their level of pleasure and psychological wellbeing (Gerdes and Mallinckrodt, 1994). Academic adjustment is a crucial element in the psychology of learning since it is closely tied to both psychological and academic wellbeing. Compared to traditional learning environments, online academic adjustment has distinctive features. However, there is not a lot of study on academic adjustment in online learning contexts. As a result, there is still much to learn about how to conceptualize academic adjustment to the online learning environment. The majority of the previous research on academic adjustment has been centered on traditional school settings. Thus, there is still much to learn about how students might succeed academically in online learning environments. The current study also intends to highlight the distinctive features of academic adjustment when studying online. We characterized academic adjustment based on the body of existing work on academic adjustment, however, as online self-regulatory learning, academic self-efficacy, educational stress, GPA, and perception of success.

Self-regulation abilities are one of the areas of academic adjustment that receive the greatest attention. As a result, it was taken into account in the current study as a significant indicator of online academic adjustment. Students who have *online self-regulation skills*, one of the most crucial markers of academic adjustment in the online environment, become aware of their level of knowledge rather than relying on teaching elements like teachers or parents to gain knowledge and skills; manage their own learning processes; and take action by actively seeking out and locating the information they need to succeed (Zimmerman, 1990; Biwer et al., 2021). When a student controls his own learning, he or she behaves responsibly, in line with his or her goals, and moves forward in their educational careers within the confines of a plan (Aydın et al., 2014). Self-regulation abilities are now more crucial than ever because of online learning, and it is anticipated that students who are scientifically literate and capable of managing their own learning processes would be able to adjust academically in this time when distant learning is still practiced. On the other hand, it is predicted that as students tried to adjust to online learning environments during the epidemic, the likelihood that they felt less capable academically, and saw themselves as less successful due to an excessive amount of homework, low academic achievement, and negative academic experiences may have increased. Indeed a systematic review on academic stress during COVID-19 has indicated that students experienced academic stress due to pile-up homework, disengagement and technical difficulties. Additionally, they found that the COVID-19 validated the signs of student mental disorders, such as physiological issues, sadness, distress symptoms, anxiety, and poor sleep, which affect their academic performance (Ibda et al., 2023). In light of this, it is considered crucial to look at the psychological processes that influence college students’ adjustment to the novel and unpredictable online learning environment during the pandemic phase where the likelihood of uncertain situations and events increases.

The students’ judgments of their own academic self-efficacy are an additional crucial indication of online academic adjustment. Academic self-efficacy refers to a student’s confidence in their ability to meet their academic obligations rather than their own skills and outlook (Millburg, 2009). Academic self-efficacy represents the belief in what one can accomplish rather than what one can do, as Rios (2002) highlights. Academic achievement and academic self-efficacy are positively correlated; as an individual’s impression of academic self-efficacy rises, so does his academic success (Millburg, 2009). Additionally, Bandura (2006) research has demonstrated that students with strong academic self-efficacy are better able to manage stress and deal with challenges. As a result, following an abrupt shift in the educational system, students’ assessments of their own abilities and strategies for dealing with difficulties play a crucial role in their ability to adjust academically to online environments.

According to Reddy et al. (2018), educational or academic stress is the psychological or physical outcome of the pressure that the student feels as a result of academic responsibilities and attitudes like succeeding in education, exhibiting high performance, worrying about the future, fulfilling what is expected of them, and homework. Similarly, Bedewy and Gabriel (2015) found that sources of academic stress are related to pressure to perform, perceptions of self-efficacy and workload for university students. Academic stress might occasionally upset the harmony between the person and his or her surroundings (Humphrey et al., 2000). According to Richlin-Klonsky and Hoe (2003), the main causes of stress are the processes of adapting to change or meeting expectations in daily routines. Beside daily stress, students may experience academic stress due to social interactions, environmental adaptation, social support, rigorous disciplinary measures, and relationship with teachers (Cheng et al., 1993). Stress also includes a triggering, exciting, and motivating component in addition to these. However, academically unmanageable stress will have a negative impact on students’ performance and learning processes (Adnan and Anwar, 2020; Mukhtar et al., 2020). Under normal circumstances, social interaction and a different environment can help reduce academic stress, but with the epidemic process, this has become more difficult. It is therefore a crucial feature of academic adaptability, especially during times of crisis.

Finally, the students’ success rate in pandemic is also an important indicator of academic adjustment in the online learning process. Research has indicated that students’ success rates are closely associated with academic adjustment (van Rooij et al., 2018). How the advantages and limitations of the online learning environment would affect students’ academic performance was in question at the beginning of the pandemic (Hollister et al., 2022; Salas-Pilco et al., 2022). The present study therefore included success rates (GPA) as an indicator of academic adaptation as well as student’s perception of their own success.

### 1.2. Intolerance of uncertainty and psychological flexibility

As stated before, university students were one of the most vulnerable groups throughout the epidemic because of the uncertainty surrounding their post-graduate lives, their work prospects, and their future anxieties and uncertainties, all of which became more pronounced with the outbreak. It is considered crucial to ascertain how much the degree of weathering the uncertainty faced by university students throughout the pandemic time is reflected in their academic adjustment for this reason. In the literature, the concept of intolerance to uncertainty is defined as a person’s incapacity to deal with uncertain conditions. It can have a substantial impact on someone’s psychological health if they are unable to handle unpredictable situations. The tendency to feel extreme fear in the face of an uncertain circumstance or occurrence that results in emotional issues is known as *intolerance of uncertainty* (Fergus and Wu, 2013). Furthermore, it is claimed that there are processes in which people have a propensity to react negatively (Starcevic and Berle, 2006; Fergus and Wu, 2013), and that the scenario is regarded as one that causes discomfort to the individual at the emotional, cognitive, and behavioral levels.

People frequently see unclear circumstances and events negatively, as a source of threat and danger (Buhr and Dugas, 2002; Dugas et al., 2005). People exhibit symptoms including tension, anxiety, rage, self-harm, and despair in response to the potential for unfavorable circumstances or events (Newman and Llera, 2011; Morriss et al., 2016; Hollingsworth et al., 2018). These newly appearing symptoms have a negative impact on positivity, and can result in inaccurate and damaging evaluation; they can produce outcomes like anticipatory and preventative anxiety (Bakioğlu et al., 2020; Satici et al., 2020b). In their research, Buhr and Dugas (2002) identified four dimensions of intolerance of uncertainty. According to Buhr and Dugas (2002), these dimensions are: (1) the individual’s experience of uncertainty is stressful and distressing; (2) uncertainties are a barrier to action; (3) the conviction that unforeseen circumstances will have a negative impact; and (4) the perception of the future’s uncertainty as injustice. It has been determined that the COVID-19 epidemic induces anxiety in all people, although the degree of anxiety varies based on the degree of control lost over one’s life, the impression of threat brought on by uncertainty, and demographic and environmental factors (Doğan and Düzel, 2020; Satici et al., 2020b; Smith et al., 2020). For instance, a recent study showed that intolerance of uncertainty is a significant risk factor for mental health issues, particularly during periods of actual physical, economic, and social unpredictability (Andrews et al., 2023). This suggests that in order to comprehend the consequences of pandemic on university students’ academic wellbeing, intolerance of uncertainty may potentially be a significant risk factor that needs to be explored.

In the literature, *psychological flexibility* is emphasized as one of the most important coping mechanisms for dealing with uncertain circumstances. According to Luoma et al. (2010), psychological flexibility is the capacity of an individual to maintain awareness of the present moment and to continue acting accordingly. It does not involve being fixated on the past or the future. The foundational idea of psychological architecture, which underpins *Acceptance and Commitment Therapy*—one of the itinerant behavioral therapy schools founded on acceptance and thought—explains all the internal processes that people go through when acting in ways that advance values without evaluating, avoiding, or changing them (Masuda et al., 2011). The acceptance and commitment method argues that the likelihood of people developing various psychopathological issues increases when psychological flexibility cannot be attained, i.e., when people have high degrees of psychological rigidity. Psychological flexibility can be conceptualized as a generalized or higher-order capacity to adapt appropriately to situational demands while pursuing longer-term goals, allowing for the choosing of coping mechanisms that are appropriate for a specific circumstance. In order to choose coping mechanisms that are appropriate for a particular situation, one must change behavior in a way that (1) includes conscious and open contact with thoughts and feelings, (2) appreciates what the situation affords, and (3) serves one’s goals and values (Hayes et al., 2011). This particular model of psychological flexibility incorporates experiential and cognitive processes in order to cope with any challenging situation (McCracken and Morley, 2014). According to research, even during the COVID-19 epidemic period, when people’s ability to control their environment is at its lowest, their psychological flexibility skills can help them cope with stress related to anxiety, depression, and pandemic (Pakenham et al., 2020; Smith et al., 2020). One of the limited numbers of studies found that psychological flexibility and intolerance of uncertainty considerably moderated the relationship between social isolation and psychological discomfort, wellbeing and valued living in COVID-19 (Smith et al., 2020). However, adults rather than university students were used in the study. To our knowledge, there have been no studies done that have examined university students’ psychological flexibility and intolerance of uncertainty in COVID-19. From the existing information on psychological flexibility and intolerance of uncertainty it may be predicted that university students who struggle to deal with uncertainty and feel less in control of their lives and futures throughout the COVID-19 period may benefit from developing their abilities of psychological flexibility. University students with high psychological flexibility skills are expected to use their online self-regulation skills much more effectively, to perceive themselves as academically more competent and capable, to achieve higher levels of success, and they are likely to experience lower levels of educational stress. Therefore, it may be argued that they can demonstrate considerably higher academic adjustment in online learning procedures. On the other hand, it’s possible that students who are unable to effectively use their psychological flexibility skills during the epidemic, when they must deal with uncertainty, struggle to use their self-regulation skills in online learning processes, believe they are less capable in the academic sense than they actually are, and as a result perform worse and feel more stressed at school.

Given all of this data from the research, it is clear that the COVID-19 pandemic’s move in teaching strategies to the online setting has had an effect on both students’ views toward learning more broadly and their capacity to adapt academically. Taking into account these restrictions, the opportunities in online education may not be equal to those in traditional classroom settings and may make it more difficult to adjust to academic and university life. These challenges associated with online learning are a substantial hindrance to students’ academic adaption to online learning environments (Okan, 2020; Tüzün and Yörük Toraman, 2021; Salas-Pilco et al., 2022). In this scenario, when university students’ power over external circumstances and events has diminished and their level of uncertainty has increased due to the pandemic, it is anticipated that it may be harder for them to concentrate on their academic tasks and responsibilities. University students with a high level of sensitivity to uncertainty exhibit higher levels of anxiety and more avoidant behavior as a result of these experiences, according to the study by Buhr and Dugas (2012). Thus, the current study’s goal is to determine the degree to which psychological flexibility and intolerance of uncertainty would influence university students’ academic adjustment.

## 2. Present study

**FIGURE 1:**
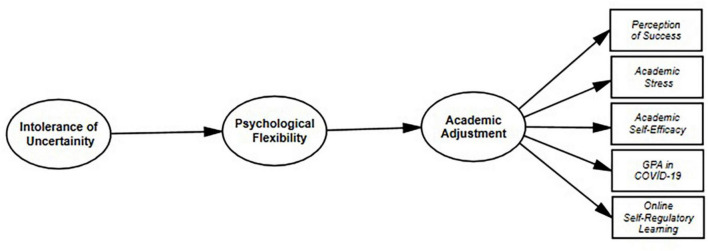
Proposed model.

In this regard, university students’ levels of psychological flexibility were incorporated in the model as a mediator variable, university students’ levels of intolerance of uncertainty as a predictor variable, and academic adjustment levels in the online learning process as an outcome variable. Namely, the study’s independent variables are university students’ levels of psychological flexibility and their intolerance for uncertainty. University students’ academic adjustment was identified as the dependent variable. The proposed model states that university students’ intolerance of uncertainty is indirectly related to their capacity for academic adjustment through their levels of psychological flexibility (see Figure 1). Thus, in our study a complete mediation model was tested. According to this model, the hypotheses of the research were established as follows:

**Hypothesis 1:** University students’ high level of intolerance to uncertainty as perceived during the COVID-19 pandemic negatively predicts their levels of psychological flexibility.

**Hypothesis 2:** University students’ levels of psychological flexibility during the COVID-19 epidemic process positively predicts their levels of academic adjustment in the online learning process.

**Hypothesis 3:** University students’ psychological flexibility levels act as a complete mediator in the relationship between their level of intolerance of uncertainty and their academic adjustment in the online learning process.

## 3. Materials and methods

### 3.1. Design

This research was designed in a relational research model in order to examine the mediating role of psychological flexibility in the relationship between intolerance of uncertainty and academic adjustment experienced by university students who continue their education online during the pandemic period. Relational studies are studies conducted to reveal the relationships between two or more variables, to understand cause-effect relationships, and to make predictions based on the relationships between variables (Frankel et al., 2011). In this context, the relationship between the variables was tested with the Structural Equation Model method and the findings were interpreted in the light of the literature. Relationships between variables can be investigated through different statistical techniques in relational studies. In this study, a model based on a theoretical basis in which the predictability of university students’ academic wellbeing during the pandemic process by their intolerance to uncertainty and psychological flexibility was directly and indirectly examined was tested through path analysis. In the model, Intolerance of uncertainty was defined as the independent variable, academic adjustment of university students was defined as the dependent variable, and psychological flexibility was defined as a mediator.

### 3.2. Participants

The research was conducted with the data obtained from a total of 384 participants (4 responses excluded as outliers), 290 female and 92 male students, 2 other between the ages of 18 and 34 (*M*_*age*_ = 21.43, *SD*_*age*_ = 2.04), located in Türkiye and continuing their university education with distance education, in the spring semester of the 2020–2021 academic year. Different online communication channels (social media platforms, classroom e-mail group, classroom WhatsApp groups, etc.) were used to reach the participants. The individuals reached by snowball sampling method were presented with a link to the data collection tools arranged through “Google Forms” and the data were collected by obtaining their explicit consent.

### 3.3. Materials

In our study, a personal information form created by the researchers was used to obtain demographic information about the participants. In addition, the Intolerance of Uncertainty Scale (BTS-12) was used for the intolerance of uncertainty variable, the Acceptance and Action Form-II was used to measure the psychological flexibility variable, and more than one measurement tool was used for the academic adjustment variable. These tools are; Educational Stress Scale, Academic Self-Efficacy Scale, Online Self-Regulatory Learning Scale and academic GPA. Psychometric information about these measurement tools is given below.

#### 3.3.1. Personal information form

The following information was collected from the participants in the form created by the researchers: Age, gender, university, department, class level, demographic characteristics such as who the student stays with, how many people they live with, study environment, access to online resources. It was assumed that there may be important variables shaping academic adaptation during the pandemic period and were included in the study.

#### 3.3.2. Intolerance of uncertainty scale (BTS-12)

In order to measure intolerance of uncertainty we used the scale developed by Carleton et al. (2007) was adapted to Turkish and Turkish culture by Sarıçam et al. (2014). The scale consists of four main factors which can be grouped into two sub dimensions: anticipatory and preventative anxiety. The Cronbach’s alpha of the 27 -item scale was 0.91, and the test–retest reliability was 0.78. It is emphasized that this scale can distinguish people with high and low anxiety levels in the non-clinical samples, therefore, criterion validity is sufficient.

#### 3.3.3. Acceptance and action form-II

In order to measure psychological flexibility we used a scale developed by Bond et al. (2011), the scale was adapted into Turkish by Yavuz et al. (2016). The scale consists of seven items and has a single factor structure. The scale, which is a Likert type, is scored with a seven-point scale (1: Never true, 7: Always true.) The Cronbach’s alpha of the scale is 0.84. It has been found that the form has good internal consistency.

#### 3.3.4. Academic adjustment scales

In our study academic adjustment is characterized by the indicators of online self-regulatory learning, academic self-efficacy, educational stress, GPA, and perception of success. The information on these measurements was presented below:

1. *Educational Stress Scale.* The scale, which was developed by Sun et al. (2011) and adapted into Turkish by Seçer et al. (2015), consists of 16 items and 5 factors. These are: “Work pressure,” “Workload,” “Grade anxiety,” “Self-Expectation,” and “Hopelessness.” The five-point Likert scale is graded between 1 (I strongly disagree) and 5 (I totally agree). Cronbach’s alpha for the scale 0.72. The Cronbach’s alpha of the scale was 0.86, and the test–retest reliability was found to be 0.81. In line with the percentages obtained, it has been concluded that it is sufficiently reliable and valid for social sciences. In our study, participants’ total scores from the scale were used for the model analysis.
2. *Academic Self-Efficacy Scale.* The scale was developed by Kandemir (2014) and consists of three factors. These are: “Self-efficacy for coping with academic problems,” “Self-efficacy for academic effort,” “Self-efficacy for academic planning.” The 27 scale is in a five-point Likert type and participants rated the items on a scale between 1 (*strongly disagree*) and 5 (*strongly agree*). The overall Cronbach’s alpha of the scale was found to be 0.92. It is possible to say that the scale is reliable and valid. Participants’ total scores from the scale were added to the model in the study.
3. *Online Self-Regulatory Learning Scale.* Developed by Barnard et al. (2009) and adapted into Turkish by Samsa-Yetik (2011), the scale consists of 24 items and 6 factors, these are: Goal Setting, Setting Environment, Time Management, Help Seeking, Task Strategies and It is Self Evaluation. The five-point Likert scale is graded between “*I strongly agree”*’ and “*I strongly disagree*.” The Cronbach’s alpha of the scale is 0.89. Accordingly, it can be said that the scale is valid and reliable. Also for this variable, the total scores of the participants were used for the model analysis.
4. *GPA.* We asked students’ the academic grade point average of the last semester before COVID-19, and their overall academic grade point average of the semester they attended in the COVID-19. In this way, we used this information to make inferences about how the courses that the student took online affected the academic grade point average.
5. *Perception of Success*. In order to measure students’ perception of success, we ask participants to answer a single item which is *“How successful do you see yourself in online courses during COVID-19?”* on a scale between *1 (not at all)* and *10 (very much).*

### 3.4. Analysis

In our study, the mediating role of psychological flexibility levels between the levels of intolerance of uncertainty and academic adjustment of university students who continued their online education during the epidemic was tested with the Structural Equation Modeling (SEM). Amos 21 program (Arbuckle, 2011) was used in the analysis. Before the main analysis, the data set was examined in terms of missing data, univariate and multivariate outliers, univariate and multivariate normality, linearity, and multicollinearity problems. In our study, χ^2^, RMSEA (Root-Mean-Square Error of Approximation), CFI (Comparative Fit Indices), AGFI (Adjusted Goodness of Fit Indices), GFI (Goodness of Fit Indices), S-RMR (Standardized Root Mean Square Residual) and NNFI (Non-normed Fit Indices) goodness of fit values were examined in order to examine the fit of the models with the data. In this direction, the following criteria were taken into account in the study of the model goodness of fit indices: RMSEA ≤ 0.10, GFI ≥ 0.90, AGFI ≥ 0.90 (Kline, 2015), CFI ≥ 0.90 (Brown, 2015), NNFI ≥ 0.90, and SRMR ≤ 0.10 (Kline, 2015).

## 4. Results

### 4.1. Descriptive statistics

The sample of the research consists of university students studying at different universities and programs in Türkiye in the 2020–2021 Fall semester. In the analysis, 290 females, 90 males and 2 other were included. The age of the participants was between 18 and 34 (*M* = 21.43, *SD* = 2.04). As seen in Table 1, 90% of participants were living with 0–4 individuals during COVID-19. Finally, almost 70% of participants did not have a study space of their own. Descriptive statistics for the sample are presented in Table 1.

**TABLE 1:** Descriptive statistics for research group.

Variable	Frequency (f)	Percentage (%)
**Sex**		
Female	290	75.5
Male	92	24.0
Other	2	0.5
*Sum*	384	
**Grade**		
Preparatory	37	9.6
1st. grade	32	8.3
2nd. grade	84	21.9
3rd. gradef	138	35.9
4th. grade	93	24.2
*Sum*	384	
**Number of people living together**		
0–4	347	90.4
5–7	35	9.2
8 and above	2	0.6
*Sum*	384	
**Study space**		
Yes	116	30.2
No	268	69.8
*Sum*	384	

### 4.2. Results for the proposed model

Information on the mean and standard deviation values of the Pearson correlation coefficients of all the variables in the hypothetical model proposed in our study are presented in Table 2.

**TABLE 2:** Pearson correlation coefficients between all variables and mean and standard deviation values of the variables.

	1	2	3	4	5	6	7	8	*X*	*S*
1. Intolerance for uncertainity	–								39.5313	9.44
2. Psychological flexibility	0.54**	–							25.4714	9.21
3. Online self-regulatory learning	0.05	0.02	–						72.7161	17.90
4. Educational stress	0.42**	0.48**	0.07	–					46.6432	11.42
5. Academic self-efficacy	−0.12**	−0.24**	0.20**	−0.21**	–				66.2422	14.52
6. Perception of success	−0.10*	−0.16**	0.16**	−0.18**	0.27**	–			6.2452	2.2471
7. GPA in the pandemic	−0.06	−0.04	−0.01	−0.06	0.15**		–		3.1453	0.5147
8. GPA before the pandemic	−0.05	−0.13**	0.080	−0.18**	0.22**	0.21**	0.72**	–	3.3687	0.4632

*p < 0.05. **p < 0.01.

As seen in Table 2, it is seen that only the online self-regulatory learning scores and GPA in the proposed model do not have a statistically significant relationship with any other variables. Apart from this, it was concluded that all the other variables were in a statistically significant relationship with each other at the intermediate level and close to the intermediate level. After examining the relationship between the variables, a measurement model was created for the latent and observed variables in the research. The measurement model was based on 2 latent variables and 8 observed variables, including intolerance of uncertainty (anticipatory anxiety and preventative anxiety), and psychological flexibility, academic adjustment (educational stress, academic self-efficacy, online self-regulatory learning, GPA, perception of success) has been tested. As a result of the analysis, the values of goodness of fit regarding the fit of the data with the model are as follows: χ^2^ (14, *N* = 384) = 125,565, *p* < 0.01, CFI = 0.81, Standardized RMR = 0.08, GFI = 0.91, AGFI = 0.81. It is seen that the values of goodness of fit are acceptable and close to perfect limits. It was concluded that all factor loads were statistically significant and ranged between 0.27 and 0.97, except for the observed variable of online self-regulatory learning and GPA in the measurement model. In line with these findings, it can be said that all observed variables, except for the online self-regulation variable and GPA, are suitable indicators for the related latent variable.

### 4.3. Results for the structural model

In line with the findings obtained from the measurement model, the online self-regulatory learning and GPA, which are two of the latent variables of academic adjustment, were removed from the model, and a structural model was created. The goodness of fit values reached according to the first analysis for the structural model: χ^2^ = 3600.502, *p* < 0.01 χ^2^/sd = 6.93, RMSEA = 0.12; NNFI = 0.82, CFI = 0.84, Standardized RMR = 0.07, GFI = 0.85, AGFI = 0.77. Initial goodness of fit values for the structural model were close to acceptable *but* partially weak. For this reason, suggested modifications for the model were reviewed. Similarly, in the original adaptation study for the AAQ, the items 1st and 4th were modified in the model (Yavuz et al., 2016). Particularly in our study, it was observed that the covariance matrix to be formed between the error variances of the 1st (*My painful experiences and memories make it difficult for me to live a life that I would value*) and 4th (*My painful memories prevent me from having a fulfilling life*) items of the psychological flexibility variable would cause the greatest decrease in the chi-square value. In this direction, the relevant items were reviewed, and it was decided that the modification proposal could be taken into account since they are very close to each other. The analysis was repeated by adding the modification to the model. Model goodness of fit values after modification: χ^2^ = 218.316, *p* < 0.01 χ^2^/sd = 4.28, RMSEA = 0.09; NNFI = 0.89, CFI = 0.91, Standardized RMR = 0.05, GFI = 0.90, AGFI = 85. With the final analysis, the model’s goodness-of-fit values were acceptable and reached perfect fit. With this result, it can be said that the model fits well with the data. With the validated model, it plays a mediating role between psychological flexibility levels, intolerance of uncertainty and academic adjustment for university students during the epidemic period. Goodness-of-fit findings regarding the structural model are presented in Table 3.

**TABLE 3:** Goodness of fit for the structural model.

Fit indexes	Criteria	Coefficients of fit of the model
χ^2^/sd	<5/1	4.28
GFI	>0.90	0.904
AGFI	>0.90	0.85
RMSEA	<0.08	0.09
S–RMR	<0.05	0.0593
CFI	>0.90	0.91
NNFI	>0.90	0.89

Bootstrap analysis was also performed to test whether the mediating role of psychological flexibility was significant. As a result of the analysis, it was concluded that the complete mediating role of psychological flexibility was statistically significant (β = 0.012, 90% CI [−0.312, −0.764]) Confidence interval values for the model are presented in Table 4.

**TABLE 4:** Confidence interval values for the proposed model.

Mediation Effects	Standardized indirect effect (β)	Bootstrap (Lower bounds/Upper bounds)% 90 CI	Hypothesis result
IoU →PF→AW	0.012	−0.312/−0.764	Supported

IoU, intolerance of uncertainty; PF, psychological flexibility; AA, academic adjustment; CI, confidence interval.

**FIGURE 2:**
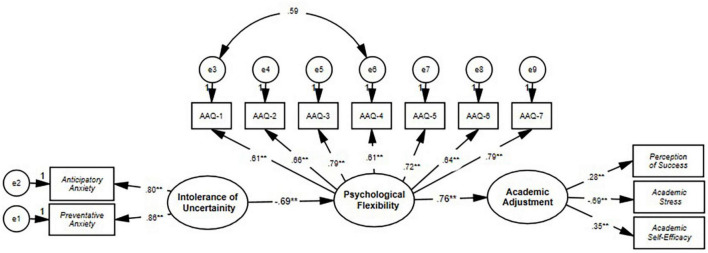
The final structural model AAQ, acceptance and action questionnaire. The numbers in the paths between the variables show the correlation coefficients between the variables. **p < 0.01.

The final model can be seen in Figure 2. The correlation between tolerance for uncertainty and psychological flexibility is negative and statistically significant (β = −0.69, *p* < 0.001). Psychological flexibility is considerably correlated with academic adjustment (β = 0.76, *p* < 0.001). In a slightly different way than we had anticipated, academic adjustment was predicted by perception of success (β = 0.28, *p* < 0.001), educational stress (β = −0.69, *p* < 0.001), and academic self-efficacy (β = 0.35, *p* < 0.001). GPA and online self-regulatory learning were not found related to academic adjustment. Possible explanations for this finding were made in the discussion below.

## 5. Discussion

In the present study, it was investigated whether psychological flexibility skills have a mediating effect on the relationship between university students’ intolerance to uncertainty levels and their academic adjustment in the online learning process during the COVID-19 pandemic. For the purposes of the study, statistical significance was investigated, and the model was found to be largely supported. In line with the findings of the study, the relationship between the academic adjustment and the intolerance level for uncertainty of university students who continued distant education during the pandemic period was predicted via the levels of psychological flexibility. Psychological flexibility is seen as a crucial quality with a protective function since it enables people to be less influenced by stressful situations or events when confronted with them (Masuda et al., 2010; Meyer et al., 2013; Kleszcz et al., 2018). There are studies showing that psychological flexibility has a protective function for individuals who try to adapt to daily life with negative emotions such as increased stress and anxiety in the COVID-19 pandemic (Satici et al., 2020a; Smith et al., 2020; Wąsowicz et al., 2021). Another study on children demonstrated that psychological inflexibility was related to depressive symptoms, poor sleep quality and adverse childhood experiences (Baojuan et al., 2023). This suggests that being psychologically flexible is a crucial skill that helps youngsters deal with challenging occurrences in life. Our findings are consistent with these earlier research and lend credence to the idea that psychological flexibility serves a protective role throughout the pandemic period. Therefore, it’s crucial to provide young people with the tools they need to grow psychologically flexible in order to boost their resilience, particularly in times of crisis.

Although the pandemic process affected all facets of society, the majority of studies examining the role of psychological flexibility were conducted with adults. In these studies, it was concluded that there is a high level of relationship between intolerance of uncertainty and psychological flexibility (Satici et al., 2020b; Smith et al., 2020; Wąsowicz et al., 2021). A sample of university students was used in our study to explore the relationship between psychological flexibility and intolerance to uncertainty, and the results showed that these two variables were strongly connected. It might be argued that this finding provides an important theoretical basis for students’ ability to operate psychologically and academically, as well as their intolerance of uncertainty and psychological flexibility in unpredictable and stressful conditions, including pandemic. There is limited research on the psychological flexibility and academic adjustment of university students. Although there haven’t been many studies, those that have accord with our findings. For instance, a study by Gadosey et al. (2022) discovered that motivation and loneliness were connected to academic satisfaction for university students in the pandemic, which suggests that psychological processes and academic adjustment are linked. According to a different study (Ventura-León et al., 2022) academic performance may be linked to depressive and anxiety symptoms in COVID-19. Given the results of these studies, it is possible to draw the conclusion that psychological flexibility is an essential skill for both prevention and intervention in college students’ academic functionality.

In our study, the relationship between university students’ level of intolerance to uncertainty and their academic adjustment over their psychological flexibility skills was examined. Academic adjustment was implicitly defined by the variables of academic self-efficacy, educational stress, online self-regulatory learning, perception of success and academic GPA. Online self-regulation learning and GPA, however, were not connected to academic adjustment. This could mean that the degree to which students perceive themselves to be successful and competent as well as the amount of educational stress are more likely to influence academic adjustment in online learning. Additionally, it’s possible that academic adjustment in online contexts differs from academic adjustment in traditional settings in certain ways. While educational stress, academic self-efficacy and perception of success had a statistically significant relationship with academic adjustment, GPA and online self-regulatory learning did not. This finding can be interpreted in a variety of ways. First of all, the online self-regulation scale used in the study measures the level of individuals’ ability to manage processes such as goal setting, environment structuring, time management, and help seeking in online environments (Samsa-Yetik, 2011). Since the scale was developed before the epidemic, it is possible that it did not meet the expectation in measuring online self-regulation processes during the COVID-19. Another reason could be that since the data collection was executed via an online form, it is possible that errors caused by participants and their environments could have affected the results.

Another variable did not yield any significant correlation with academic adjustment was academic GPA. The reason why GPA did not correlate with academic adjustment could be that academic success might not be impacted by COVID-19. Indeed, in our study, we found that pre-pandemic GPA is positively and highly correlated with post-pandemic GPA (*r* = 0.72, *p* < 0.001). In fact, GPA is frequently employed as a traditional indication of academic adjustment (van Rooij et al., 2018). For the educational system, COVID-19 is a novelty. Other elements might have affected how well students adjusted. This view is also supported by our research, which indicates that academic performance appears to be less relevant than stress and the appearance of accomplishment.

When the limited number of studies conducted are examined, it is seen that different results have been found regarding the academic performance of university students throughout the pandemic. The first line of research suggests that there is a negative impact of COVID-19 on academic performance. Especially in this process, there are studies that have found that the fact that students have to cope with many different obstacles at the same time because they try to keep up with the online learning processes with the least information and support affects their academic performance (Limniou et al., 2021; Mahdy and Ewaida, 2022). It is possible for students to experience negative academic experiences such as feeling less academically competent, feeling less successful due to excessive workload, and low academic success while trying to adapt to online learning environments during the pandemic process. On the other hand, the second line of research argues that COVID-19 pandemic, students’ learning strategies turned into a more continuous habit and that increasing their productivity had a positive effect on their learning performance (González-Sanguino et al., 2020). For instance there are studies indicating that some students are more comfortable when asking questions and participating in discussions in online learning environments (Hollister et al., 2022). This might increase their academic engagement which might be related to increased GPA. Moreover, there are findings that the academic performance of students during the pandemic may increase compared to before due to reasons such as stretching the distance education processes during the pandemic process, softening the evaluation criteria due to unusual pandemic conditions, for instance, performance evaluation with alternative methods such as homework and projects instead of exams (Okan, 2020; Serçemeli and Kurnaz, 2020; Özlem and Kizilcalioğlu, 2020; Tüzün and Yörük Toraman, 2021). There are also findings that socioeconomic level is one of the important criteria that is thought to be effective on the academic performance of students during the pandemic process. For example, in a large-scale study conducted by Rodríguez-Planas (2022) with 12 thousand students, it was stated that especially high-achieving but low-income students both lost their academic performance and the course credits they received during the pandemic process. Again, it was concluded that the academic performance of low-performing and low-income students was more negatively affected during the pandemic process than high-income students. Finally, the third line of research has no significant association between academia performance and COVID-19 period (Roberts et al., 2023). Therefore, the direction of the COVID-19 on academic performance is so far inconclusive. More research on the processes of academic performance in the times of crisis should be conducted.

According to the different findings on students’ academic adjustment, it demonstrates that when stressful life events with widespread repercussions, such as pandemics, are experienced, it is important to explore in greater detail the psychological and social processes that support students’ academic functionality. For this reason, this study contributes to explain the psychological processes that contribute to the academic adjustment of students. From our findings, it was concluded that while educational stress and academic self-efficacy perceptions were an important indicator as a sign of academic adjustment, online self-regulation was not a significant indicator of academic adjustment during the pandemic process. Therefore, it appears that future research, particularly using online processes, would be crucial especially in a time of growing unpredictability. Furthermore, examining the academic and psychological mechanisms for students at different learning levels will also contribute to the literature. As we found in our study, intolerance of uncertainty and psychological resilience provide an important theoretical framework in stressful times. Taking into account together with other studies in the literature, it is thought that it is important for future studies to examine how this framework contributes to the functionality of individuals in different life areas. Finally, investigating how psychological flexibility works in different socioeconomic groups during the processes of increasing uncertainty will deepen the literature.

There are some limitations to our study. It was conducted right as the pandemic was just getting started. As a result, the study’s measures weren’t entirely updated. This emphasized certain restrictions on our study. However, the conditions surrounding the outbreak and lockdown at the time were uncertain, and we had no idea how long it would undergo. As a result, we had little time to complete our research. We made the decision to proceed forward with the measures that at the time best supported our hypotheses.

## 6. Conclusion and implications

Our findings suggest that psychological flexibility in times of stress is strongly related to academic adjustment. Although there are many studies on psychological flexibility with adults, there are still few with young people. It is therefore vital to conduct further research on the psychological adaptability of university students under pressure. Examining the relationship between psychological health and academic adjustment is more crucial than ever in light of the current global crisis. This study is important because it sheds light on how a crucial aspect affects university students’ academic success in COVID-19. The findings highlight the need for additional study on young adults’ psychological flexibility. Studies exploring the psychological adaptability of various groups, for instance, might add to the body of knowledge. An intersectional perspective on psychological adaptability would also significantly supplement the available data. We already know from intersectional studies how our various identities combine to produce a unique experience for all (Crenshaw, 2013). There is a growing body of research that has indicated that multiple identities are related to psychological wellbeing and coping mechanisms (Seaton et al., 2010; Settles and Buchanan, 2014; Brown and Moloney, 2019). Therefore, it would be also crucial to examine how a person’s ethnicity, color, gender, age, or disability may affect their coping mechanisms under stressful times, such as during a pandemic.

According to positive psychology, everyone has the ability to accomplish goals and adjust to a variety of challenging circumstances (Yates and Masten, 2004). But in emergency situations, they might not always be or be easy to find. Therefore, it would be advantageous to plan research that looks at how to unlock young people’s full potential for resilience and flexibility in difficult circumstances. For example, the capacity for mindfulness, emotional control, resiliency, hope, and psychological need satisfaction would be essential psychological traits to overcome challenges. Additionally, there are a few other constructs related to psychological flexibility in the literature (acceptance, cognitive defusion, flexible present-focused attention, self-as-observer, values, and committed action), which researchers may use to further explore these characteristics in the context of crises (McCracken and Morley, 2014).

The five sub-dimensions of academic adjustment in our study were educational stress, GPA, self-efficacy, perception of success, and online self-regulatory learning. Online self-regulatory learning was supposed to show a relationship with other factors, however, no meaningful connections with the other variables were found. New strategies for crisis self-regulation in online learning environments would be a significant advancement in light of this study. Additionally, the concept and components of academic adjustment would be revised. Other significant components of academic adjustment might be more applicable to online learning. Studies that were done in conventional, face-to-face learning situations have verified the majority of structures of academic adjustment and wellbeing. However, at the time our study was conducted, both students and teachers were unfamiliar with remote learning, so there were no up-to-date scales available. Therefore, it is crucial to create new scales to assess academic adjustment under conditions that change.

The findings of the current study demonstrate the importance of psychological resilience as a skill for college students to have in difficult circumstances. Therefore, university students should have psychological flexibility prioritized in prevention and response measures designed expressly for crisis situations. Additionally, psychological flexibility modules would be incorporated into educational systems to assist students who are learning remotely. A system’s flexibility is closely correlated with the adaptability of its constituent parts. Because of this, research should be done to determine how varying degrees of system flexibility would impact people. Systems would also be set up in a way that would make its constituents more resilient. According to research, social factors like group identification may also promote resiliency through higher life satisfaction in the event of an external threat, such as COVID-19 (Pagliaro et al., 2013). Therefore, it is worthwhile to investigate further how emphasizing social identity could help people establish group-based resilience in the midst of a catastrophe. Given that ongoing and potential geopolitical, climatic, and health concerns will probably result in continued exposure to severe uncertainty, the results indicate the need to investigate strategies for fostering resilience in people who find it difficult to cope with uncertainty. Future research therefore can focus on developing group based resiliency interventions which consider both individual and group level factors.

Finally, in order to promote students’ development of psychological flexibility and their academic adjustment, it is crucial to structure university-based psychological counseling facilities or educational programs in this way. In a time when threats from epidemics and worldwide crises are rising constantly, it is believed that psychoeducational procedures should be centered on fostering the psychological flexibility of university students, who are the foundation of our future.

## Data availability statement

The raw data supporting the conclusions of this article will be made available by the authors, without undue reservation.

## Ethics statement

The studies involving humans were approved by the Ankara University Ethics Committee. The studies were conducted in accordance with the local legislation and institutional requirements. Written informed consent to participate in this study was provided by the participants.

## Author contributions

EsmD: Conceptualization, Data curation, Formal analysis, Funding acquisition, Investigation, Methodology, Project administration, Software, Supervision, Validation, Writing – original draft. KS: Conceptualization, Data curation, Funding acquisition, Investigation, Methodology, Writing – original draft. EsrD: Formal analysis, Investigation, Methodology, Resources, Validation, Visualization, Writing – original draft, Writing – review and editing.
